# Infill Pattern-Dependent Mechanical Properties and In Vitro Release Behavior of FDM 3D-Printed Resveratrol Amorphous Solid Dispersion Matrix Tablets

**DOI:** 10.3390/polym18121531

**Published:** 2026-06-19

**Authors:** Lianghao Huang, Kai Zheng, Xiaofeng Chen, Yunping Zhao, Tiantian Yang, Hang Yu, Wei Zhao, Xia Zhao, Jiaxiang Zhang

**Affiliations:** 1 Key Laboratory of Marine Drugs, Ministry of Education, School of Medicine and Pharmacy, Ocean University of China, Qingdao 266003, China; 2 Pharmaceutical Products Research and Development Center, Marine Biomedical Research Institute of Qingdao, Qingdao 266137, China; 3 Shandong SMA Pharmatech Co., Ltd., Zibo 255086, China

**Keywords:** programmed drug release, 3D printing, infill patterns, hot-melt extrusion, resveratrol

## Abstract

Resveratrol (RSV) is a poorly water-soluble polyphenolic compound with various potential health benefits, but its pharmaceutical application is limited by low aqueous solubility and poor oral bioavailability. Additive manufacturing (AM), particularly fused deposition modeling (FDM) 3D printing, offers a flexible approach for fabricating oral dosage forms with customized geometry and internal architecture. In this study, hot-melt extrusion (HME) combined with fused deposition modeling (FDM) 3D printing was used to prepare RSV-loaded tablets with different infill patterns. Hydroxypropyl methylcellulose acetate succinate and hydroxypropyl cellulose were selected as polymeric carriers to prepare RSV-loaded filaments suitable for FDM printing. The effects of infill pattern on the solid-state characteristics, dimensional accuracy, mechanical properties, floating behavior, and in vitro drug release of the printed tablets were systematically investigated. Differential scanning calorimetry, powder X-ray diffraction, and polarized light microscopy indicated that RSV was mainly converted into an amorphous or molecularly dispersed state after HME and FDM processing. All designed tablets were successfully printed and showed acceptable shape fidelity, while different infill patterns resulted in variations in tablet weight, mechanical strength, floating duration, and release behavior. In vitro dissolution studies showed that the RSV release profiles were dependent on the internal infill architecture. Tablets with more complex infill patterns generally exhibited slower drug release, which may be related to differences in internal pore structure, medium penetration pathways, matrix hydration, and diffusion distance. Release kinetic analysis further suggested that RSV release from the printed tablets involved a combination of diffusion and polymer relaxation processes. These results demonstrate that infill pattern is an important structural parameter for modulating the mechanical performance and drug release behavior of FDM 3D-printed RSV tablets. This study provides useful guidance for the design of 3D-printed oral dosage forms with tunable release characteristics.

## 1. Introduction

Conventional pharmacotherapy often follows a ‘one-size-fits-all’ approach, although interindividual differences in physiology, metabolism, and disease state can lead to variable efficacy and safety outcomes [1,2,3,4,5]. These limitations are particularly relevant for chronic and complex diseases, where inappropriate exposure may reduce therapeutic benefit and increase healthcare burden [6]. Precision medicine aims to match therapeutic strategies with individual patient characteristics, disease mechanisms, and molecular profiles [7,8]. For oral drug delivery, this concept requires dosage forms that can be customized not only in dose but also in release rate and release duration, thereby improving therapeutic efficacy while reducing safety risks [9].

Several advanced drug-delivery technologies, including nanoparticles, liposomes, and microneedles, have improved the control of drug release and targeting [10,11,12,13,14]. However, many of these platforms remain difficult to individualize rapidly at the point of care. Three-dimensional (3D) printing, also known as additive manufacturing, constructs objects layer by layer from digital models [15,16], and provides a flexible manufacturing route for patient-specific dosage forms [17,18,19,20]. The first FDA-approved 3D-printed tablet, Spritam^®^ (levetiracetam), was produced using ZipDose^®^ technology to generate highly porous tablets that disintegrate rapidly in liquid, mainly addressing swallowing difficulties rather than individualized release programming. Since then, pharmaceutical 3D printing has attracted increasing attention because digital design enables rapid adjustment of formulation geometry, dose, and performance [21]. Common pharmaceutical 3D-printing techniques include stereolithography, selective laser sintering, fused deposition modeling (FDM), material extrusion, material jetting, binder jetting, and related approaches [22,23]. Among these techniques, FDM, a material-extrusion-based approach, has been widely used in pharmaceutical research because of its relatively low cost, simple workflow, and compatibility with thermoplastic polymers [24,25].

Previous FDM studies have shown that drug release can be modulated by adjusting tablet shape, size, shell thickness, internal structure, and infill density [18,19,20,26,27]. Therefore, the novelty of the present work is not the general observation that 3D-printed structures affect dissolution, but the attempt to compare several internal infill patterns under fixed external dimensions, nominal dose, shell settings, and formulation composition. This design reduces, although does not fully eliminate, confounding effects from tablet mass, surface area, and polymer-matrix hydration. Infill pattern was selected as the key variable because it can alter pore connectivity, load-bearing pathways, and diffusion routes without changing the intended dose. A cautious structure–property–release analysis is needed to guide rational pattern selection and reduce empirical trial-and-error development, which is consistent with the broader objective of integrating digitally designed dosage forms into personalized drug-delivery systems [28].

Resveratrol (RSV, 3,5,4’-trihydroxystilbene) is a natural polyphenolic stilbene found in grapes, berries, peanuts, and other plant-derived foods [29]. RSV exists as cis and trans isomers, with the trans form generally considered more abundant and pharmacologically active [30]. It has been reported to exhibit antioxidant, anti-inflammatory, anti-aging, anticancer, antidiabetic, neuroprotective, and antiviral activities [31,32,33,34,35,36,37,38,39,40,41,42]. Despite these promising biological effects, RSV has poor aqueous solubility and limited oral bioavailability. Moreover, excessive exposure may be associated with safety concerns, including DNA damage, cell death, and organ toxicity [35]. Therefore, RSV is a suitable model compound for evaluating dosage forms that combine solubility enhancement with controllable drug release.

**Figure 1:**
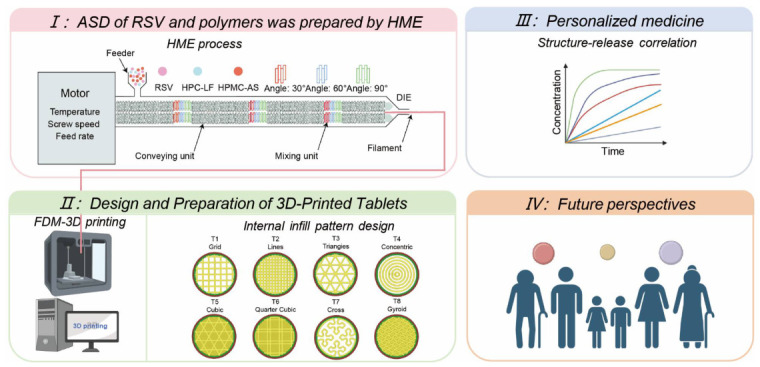
Schematic diagram of the preparation process and application of personalized pharmaceutical formulations based on HME and FDM-3D printing. Colors in the figure are used to distinguish formulation components, printing parameters, and personalized design features: RSV, HPC-L, and HPMC-AS are represented by different colors in the HME process; the colored filament segments indicate different drug/polymer compositions or deposited layers; the colored patterns in the tablet cross-sections illustrate different internal infill designs; and the multicolored release curves represent different structure–release profiles. The different colored human icons indicate patient-specific variability and the potential for individualized dosing.

To the best of our knowledge, few studies have systematically isolated the role of internal infill geometry in FDM-printed amorphous solid dispersion tablets while maintaining an identical drug–polymer matrix, external dimensions, nominal drug loading, shell configuration, and printing parameters. In this study, resveratrol was used as a poorly water-soluble model compound, and an HPMC-AS/HPC-EF-based polymeric matrix was developed through hot-melt extrusion and FDM printing (Figure 1). Unlike previous studies that primarily varied formulation composition, drug loading, or tablet size, this work focuses on infill geometry as a digital design variable to modulate tablet mechanical anisotropy and in vitro release behavior within the same polymer matrix. By comparing multiple infill architectures under controlled formulation and dimensional conditions, this study provides a clearer structure–property–release relationship for polymer-based printed tablets and demonstrates how matrix–geometry coupling can be used as a formulation-independent strategy for tuning the performance of FDM-printed oral dosage forms.

## 2. Materials and Methods

### 2.1. Materials

Resveratrol (RSV) was purchased from Macklin Biochemical Co., Ltd. (Shanghai, China). Hydroxypropyl methylcellulose acetate succinate (HPMC-AS; grade: H grade) was purchased from Taian Ruitai Cellulose Co., Ltd. (Taian, China). Hydroxypropyl cellulose (HPC-EF) was kindly provided by Ashland (Shanghai, China). Soluplus^®^, Plasdone K25, Plasdone K90, Kollidon^®^ VA64, and Kollidon^®^ SR were purchased from BASF (Ludwigshafen, Germany). All other chemicals, solvents, and reagents were of analytical or HPLC grade.

### 2.2. Methods

#### 2.2.1. Formulation Studies

##### Preliminary Formulation Screening

RSV and polymers were dried in a vacuum oven at 50 °C for 24 h before use. Physical mixtures (PMs) containing 20% (*w*/*w*) RSV and 80% (*w*/*w*) polymer (Table S1) were prepared by manual blending. Before filament extrusion, the mixtures were processed using an NLH-600 CG heated hydraulic press (Tianjin Nuolei Xinda Technology Co., Ltd., Tianjin, China) to evaluate the miscibility of RSV with different polymers and the potential formation of ASDs. The pressing temperature was set to 200 °C, and each PM was heated for 5 min under an applied force of approximately 100 N. The resulting samples were ground, passed through a 40-mesh sieve, and stored in a validated desiccator until further analysis.

##### Equilibrium Solubility Measurement

The equilibrium solubility of RSV formulations listed in Table S1 was measured in simulated intestinal fluid (SIF, pH 6.8) at 37 ± 0.5 °C. Excess sample was added to 20 mL of SIF and stirred at 200 rpm for 24 h. The vials were then left undisturbed for an additional 24 h to allow undissolved particles to settle. The supernatant was carefully collected, diluted as required, and analyzed by HPLC. Each experiment was performed in triplicate (*n* = 3). Statistical significance was evaluated using one-way analysis of variance followed by Tukey’s post hoc test in GraphPad Prism 9.4 (San Diego, CA, USA).

##### HPLC Quantification of RSV

RSV was quantified using an Agilent 1260 Infinity II HPLC system (Santa Clara, CA, USA) equipped with a UV detector, and data were processed using Agilent DAD software (Version C.01.07 SR2 [255] (software version number), Agilent Technologies, Inc., Santa Clara, CA, USA). A 20 μL aliquot was injected onto a C18 Diamonsil^®^ Plus column (5 μm, 250 × 4.6 mm; DiKMA^®^, Beijing, China). The mobile phase was methanol/0.1% glacial acetic acid solution (50:50, *v*/*v*), delivered at 1.0 mL/min [33]. The detection wavelength was 308 nm, and the column temperature was maintained at 30 °C. Data are presented as the mean ± standard deviation of three independent experiments and were analyzed using Microsoft Excel (version 2310 build 16.0.16924.20054).

##### Preparation of Filaments

**Figure 2:**
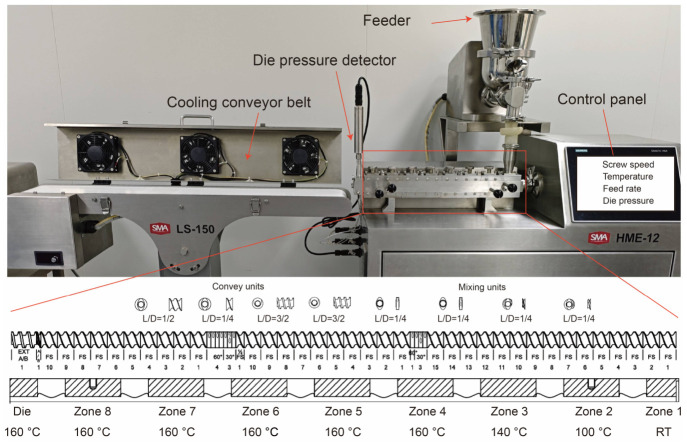
Screw configuration and extrusion setup used for filament preparation.

RSV-loaded filaments were prepared using an HME-12 twin-screw extruder (Shandong SMA Pharmatech Co., Ltd., Zibo, China) equipped with eight heating zones and a 2 mm round die. The filament formulations are listed in Table 1. The PM was manually fed into the feeding zone at approximately 0.4 kg/h, and the screw speed was set to 130 rpm. The screw configuration and extrusion temperature profile are shown in Figure 2. Temperature, torque, and die pressure were monitored in real time during extrusion. Filaments were collected using a conveyor belt only after torque and die pressure had stabilized. The use of the conveyor belt provided a relatively constant drawing speed, which helped maintain uniform filament diameter during collection. Filament diameter was monitored using a caliper to ensure suitability for FDM printing. The same batch of RSV-loaded filament was used for all subsequent FDM printing experiments to minimize batch-related variability. A portion of the extruded filaments was milled and passed through a 40-mesh sieve; this milled extrudate was denoted as EXT. All filaments and powders were stored in a validated desiccator before testing and printing.

##### Mechanical Characterization of Filaments

The flexibility and brittleness of the extruded filaments were evaluated using a TA-XT2 texture analyzer (Texture Technologies Corporation, New York, NY, USA) equipped with a TA-92N mini three-point bending fixture with a 15 mm support span. Filaments were cut into 20 mm segments and positioned on the fixture. The pre-test, test, and post-test speeds were 10, 1, and 10 mm/s, respectively. The probe was operated in distance mode and applied force over a 15 mm travel distance after contacting the filament. Breaking distance and load force were recorded and analyzed using Exponent software 6.2 (Stable Micro Systems, Godalming, Surrey, UK).

#### 2.2.2. Solid-State Characterization

##### Thermogravimetric Analysis (TGA)

The thermal degradation behavior of RSV, HPC-EF, HPMC-AS, and PM was analyzed using a Netzsch TG 209 F3 TGA (NETZSCH-Gerätebau GmbH, Selb, Germany). Samples of 2–5 mg were placed in the sample pan and heated from 20 °C to 350–400 °C at a rate of 20 °C/min under a continuous flow of ultra-pure nitrogen. Microsoft Excel (version 2310, build 16.0.16924.20054) was used to collect and analyze the data.

##### Differential Scanning Calorimetry (DSC)

The thermal behavior of RSV, HPMC-AS, HPC-EF, the PM, and the EXT was analyzed using a NETZSCH DSC 200 F3 instrument (NETZSCH-Gerätebau GmbH, Selb, Germany). Samples of 5–10 mg were sealed in standard aluminum pans and heated from 20 °C to 300 °C at 20 °C/min. Data were collected and analyzed using Microsoft Excel (version 2310, build 16.0.16924.20054).

##### Powder X-Ray Diffraction (PXRD)

The crystallinity of RSV, HPMC-AS, HPC-EF, the PM, and the EXT was analyzed using a D/max-2200PC benchtop PXRD instrument (Rigaku Corporation, Tokyo, Japan). Samples were loaded into sample holders and scanned over a 2θ range of 5–50° at 2°/min, with a step size of 0.02° and a resolution of 0.0025°. The instrument was operated at 45 kV and 15 mA. Data were collected and analyzed using Microsoft Excel (version 2310, build 16.0.16924.20054).

##### Hot-Stage Polarized Light Microscopy (PLM)

Melting behavior and residual crystalline RSV were examined using a CX40P polarized light microscope (Ningbo ShunYu Analytical Instrument Co., Ltd., Yuyao, China) equipped with a hot stage (Linkam Scientific Instruments Ltd., Salfords, UK). Drug and milled samples were evenly spread on glass slides, gently covered with coverslips, and observed at 10× magnification. Samples were heated from room temperature to the melting region at 20 °C/min. Images were captured using a digital camera (EP-SUF880, Markham, ON, Canada) with a 530 nm compensator (U-TP530, Olympus Corporation, Tokyo, Japan) under bright-field and polarized-light conditions.

#### 2.2.3. 3D Design and Printing

**Figure 3:**
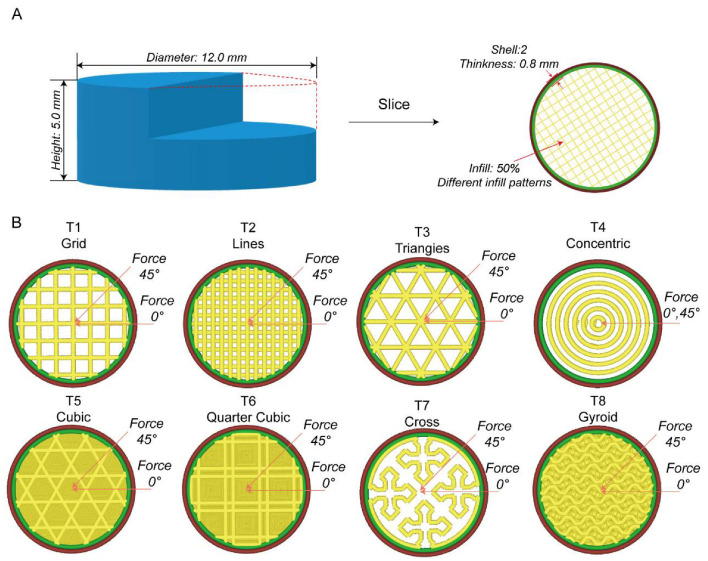
Digital tablet design (**A**) and representative internal infill patterns used for FDM printing (**B**). Colors are used to distinguish design elements: blue represents the external tablet model, yellow represents the internal infill patterns, green/red outlines indicate the shell or perimeter layers, and red arrows/lines indicate slicing, shell thickness, or force-loading directions.

Cylindrical tablets with a diameter of 12.0 mm and a height of 5.0 mm were designed using 3D Builder software (version 18.0.1931.0, Microsoft Corporation) and sliced using Cura software (version 5.2.1, Ultimaker). As shown in Figure 3, the digital models were assigned different internal infill patterns while maintaining identical external dimensions. All designs had open top and bottom structures. Tablets were printed using an Ender-3 S1 Pro FDM printer (Shenzhen Creality 3D Technology Co., Ltd., Shenzhen, China) equipped with a 0.4 mm nozzle. The nozzle temperature and build-plate temperature were set to 220 °C and 50 °C, respectively. The key printing parameters were as follows: printing speed, 50 mm/s; infill density, 50%; and layer height, 0.12 mm. EXT and PM controls were filled into capsules at approximately 410 mg per capsule. The diameter and thickness of the printed tablets were measured using a VWR^®^ digital caliper (VWR^®^, Avantor, Radnor, PA, USA), and the tablet structures were imaged using a Dino-Lite optical microscope (AnMo Electronics Corporation, New Taipei City, Taiwan).

#### 2.2.4. Hardness

The compressive resistance of the 3D-printed tablets under 0° and 45° loading orientations (Figure 3) was evaluated using a CMT 6104 testing machine (MTS Systems (China) Co., Ltd., Shenzhen, China). The loading orientation was defined as the angle between the designated reference axis of the tablet and the vertical compression direction. For the 0° orientation, the tablets were positioned directly on the lower platen according to the predefined reference direction. For the 45° orientation, the tablets were rotated to 45° relative to the compression/loading direction before testing, while the instrument and platen orientation remained unchanged. The compression speed was set to 5 mm/min using the instrument control program. Each tablet was placed on the lower platen, and testing was initiated after the upper platen made initial contact with the sample surface. The instrument was calibrated according to the manufacturer’s instructions before testing. Force-displacement data were recorded continuously for subsequent analysis of mechanical response and failure behavior. Mechanical testing was performed in triplicate for each infill pattern and loading orientation. For clarity, one representative force–displacement curve was shown for each condition, while the quantitative mechanical parameters, including maximum stress and compressive modulus, were calculated from three independent measurements and are presented as mean ± SD.

#### 2.2.5. Quantitative Analysis of Infill Structures

Quantitative structural descriptors of the FDM-printed tablets were obtained from top-view optical microscopic images. The internal infill region was defined by excluding the external shell region from the measured tablet radius. According to the tablet design, the shell thickness was 0.8 mm. Therefore, the internal infill radius ($T_{in}$) was calculated by subtracting the shell thickness from the measured tablet radius:

$$T_{in}=\frac{D}{2}-0.8$$

where D is the measured tablet diameter. The projected internal infill area ($A_{infill}$) was then calculated as:

$$A_{infill}=\pi r_{in}^{2}$$

where $r_{in}$ is the internal infill radius after excluding the external shell region.

The optical images were calibrated using the scale bar and analyzed using ImageJ (version 1.53t; National Institutes of Health, Bethesda, MD, USA). The visible open pores connected to the external environment were segmented by thresholding after conversion to 8-bit grayscale images. The top-view accessible pore fraction ($F_{top}$) was calculated as:

$$F_{top}=\frac{A_{accessible\;pore}}{A_{infill}}\times 100\%$$

where $A_{accessible\;pore}$ is the projected area of visible pores accessible from the tablet surface and $A_{infill}$ is the projected internal infill area. The accessible pore area was further calculated by multiplying the accessible pore fraction by the internal infill area. Because this analysis was based on two-dimensional top-view optical images, the obtained values were used as projected structural descriptors rather than true three-dimensional porosity. These parameters were used to compare the relative openness and surface accessibility of different infill architectures.

#### 2.2.6. Determination of RSV Recovery After FDM 3D Printing

To verify the RSV content after FDM 3D printing, a recovery experiment was performed using the FDM-processed samples. The printed filament/tablet sample was accurately weighed and dissolved to obtain a target RSV concentration of 50 μg/mL. The sample solution was prepared until no visible particles remained. The RSV concentration was then determined using HPLC. The recovery percentage was calculated as the ratio of the experimentally measured RSV concentration to the theoretical RSV concentration. The experiment was performed in triplicate, and the results are presented as mean ± SD.

#### 2.2.7. In Vitro Drug Release

Drug release from FDM-printed tablets with different internal patterns was evaluated in vitro. PM and EXT controls were filled into capsules for comparison. Each sample group contained approximately 80 mg of RSV. Dissolution testing was performed using a Chinese Pharmacopoeia paddle apparatus (RC8MD, TIANDA TIANFA, Tianjin, China) under enzyme-free simulated intestinal fluid (SIF, pH 6.8) conditions. Because HPMC-AS is a pH-dependent enteric polymer, this single-stage test was designed to compare intestinal-stage release from different internal geometries rather than to simulate the complete gastrointestinal transit process. The dissolution medium volume was 900 mL, the temperature was maintained at 37 ± 0.5 °C, and the paddle speed was 100 rpm. Aliquots were collected at 0.5, 1, 2, 4, 6, 8, 12, 16, 20, and 24 h. RSV release was quantified by HPLC using the method described in Section HPLC Quantification of RSV. After 24 h, the printed tablets were removed immediately, photographed to document swelling, and imaged using a Dino-Lite optical microscope to observe structural changes.

#### 2.2.8. Release-Kinetic Analysis

The dissolution data of the FDM-printed tablets were fitted using five mathematical models: zero-order, first-order, Higuchi, Korsmeyer–Peppas, and Peppas–Sahlin models (Table 2). These models were used to compare release behavior among tablets with different internal patterns. The fitted parameters were interpreted descriptively because model fitting alone cannot prove a unique release mechanism in non-disintegrating, swelling polymer matrices. The goodness of fit was evaluated using the correlation coefficient (R^2^).

#### 2.2.9. Statistical Analysis

All quantitative data are presented as mean ± standard deviation (SD), and the number of replicates is indicated in the corresponding figure captions or table notes. Unless otherwise stated, the replicates were independently prepared samples. Statistical analysis was performed for the solubility study using ANOVA followed by multiple-comparison tests. A value of *p* < 0.05 was considered statistically significant. For representative images and solid-state characterization results, including TGA, DSC, PXRD, and PLM, statistical analysis was not performed because these experiments were used for qualitative characterization.

## 3. Results and Discussion

### 3.1. Formulation and Process Development

#### 3.1.1. Calibration and Validation of RSV Quantification

Standard RSV solutions were prepared at concentrations of 5, 25, 50, 100, 200, and 300 μg/mL. The HPLC conditions described in Section HPLC Quantification of RSV were used to generate the calibration curve. The method showed linearity over the tested concentration range (R^2^ = 0.9915). As shown in Figure S1, an RSV solution with a nominal concentration of 150 μg/mL was analyzed and gave a deviation of less than 0.5% from the calculated value, supporting the suitability of the calibration curve for subsequent quantification.

#### 3.1.2. RSV Recovery After FDM 3D Printing

To confirm whether RSV content was maintained after thermal processing, the RSV recovery of the FDM-processed samples was determined by HPLC. The FDM-processed sample was accurately weighed and dissolved to prepare a solution with a target RSV concentration of 50 μg/mL. After complete dissolution with no visible particles, the RSV concentration was quantified using the validated HPLC method. As shown in Figure S2, the RSV recovery was approximately 100%, indicating that no obvious RSV loss occurred during FDM 3D printing. This result suggests that the selected FDM processing condition did not cause detectable RSV content loss under the current analytical conditions.

#### 3.1.3. Solubility of RSV, PMs, and Extrudates

**Figure 4:**
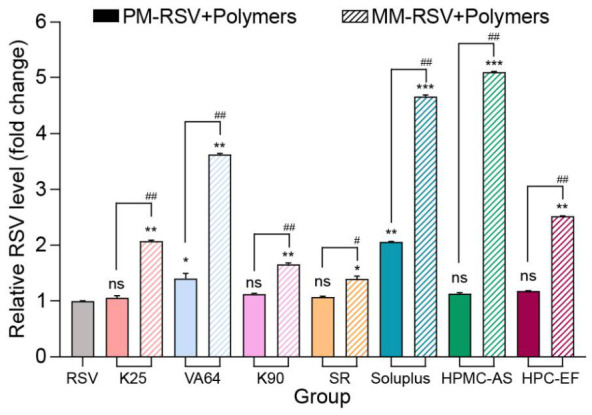
Relative RSV solubility of formulations prepared by physical mixing (PM) and melt processing (MM) in pH 6.8 medium at 37 °C. Data are presented as mean ± SD. Statistical analysis was performed using ANOVA with multiple-comparison tests. * *p* < 0.05, ** *p* < 0.01, and *** *p* < 0.001 vs. crystalline RSV; ^#^ *p* < 0.05 and ^##^ *p* < 0.01 vs. the corresponding PM group; ns, not significant.

The solubility-enhancing effects of different polymers and melt-processed samples were evaluated in pH 6.8 medium. The intrinsic solubility of crystalline RSV was 50.6 μg/mL. Most PM formulations produced only limited improvement in RSV solubility; Soluplus^®^ was the exception, increasing the measured solubility to 104.3 μg/mL (Figure 4). This increase may be related to the ability of Soluplus^®^ to form micellar structures and inhibit recrystallization in supersaturated solutions. After melt processing, RSV solubility increased markedly to 236.2 μg/mL with Soluplus^®^, 127.6 μg/mL with HPC-EF, 183.6 μg/mL with Kollidon^®^ VA64, and 258.4 μg/mL with HPMC-AS. Although HPMC-AS, Soluplus^®^, and Kollidon^®^ VA64 all showed strong solubilization potential, filaments containing high proportions of Soluplus^®^ or Kollidon^®^ VA64 have been reported to exhibit poor mechanical performance, which may interrupt FDM printing [43]. Therefore, HPMC-AS was selected primarily because it provided high apparent RSV solubility after melt processing and could form printable filaments when combined with HPC-EF. The rationale should not be interpreted as evidence that HPMC-AS is required to protect RSV from gastric acid or to reduce severe side effects. Instead, HPMC-AS served as a pH-dependent model matrix for evaluating intestinal-stage, geometry-regulated release. This selection also imposes a formulation limitation: compared with fast-dissolving polymers, an enteric HPMC-AS matrix can delay and restrict RSV release, and the dissolution threshold may depend on the specific HPMC-AS grade [44,45].

#### 3.1.4. Mechanical Properties of Filaments

**Figure 5:**
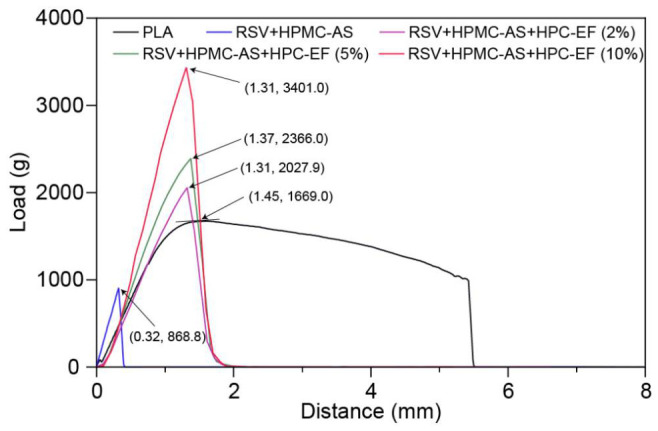
Three-point bending load–distance curves of commercial PLA, RSV/HPMC-AS, and RSV/HPMC-AS/HPC-EF filaments containing 2%, 5%, or 10% HPC-EF. The annotated coordinates indicate the breaking distance (mm) and corresponding load at break (g) for each filament.

Because FDM printing requires filaments with sufficient flexibility and mechanical strength, HME-prepared filaments were evaluated using a three-point bending test [46]. As shown in Figure 5, the RSV/HPMC-AS filament without HPC-EF was brittle, with a loading force of 868.8 g and a breaking distance of 0.32 mm. The high melting point and rigid crystalline structure of RSV may increase filament brittleness by affecting the mechanical response of the RSV/HPMC-AS system [47]. Potential hydrogen-bonding interactions between RSV and HPMC-AS may also contribute to the observed behavior [48,49]. Adding HPC-EF at 2%, 5%, and 10% progressively increased the load at break to 2027.9, 2366.0, and 3401.0 g, respectively, all exceeding that of commercial PLA under the same testing conditions. The breaking distance of the HPC-EF-containing filaments ranged from 1.31 to 1.37 mm, indicating improved processability but no clear concentration-dependent increase in flexibility. To further assess their suitability for downstream FDM processing, the filaments were also subjected to a preliminary printability evaluation. Filaments containing less than 5% HPC-EF frequently fractured during printer feeding and preliminary printing, whereas 10% HPC-EF enabled continuous printing without breakage. Therefore, the formulation containing 20% RSV, 70% HPMC-AS, and 10% HPC-EF was selected for the preparation of printed tablets because it provided the highest load at break, acceptable bending displacement, stable filament feeding, and continuous printability among the tested formulations.

### 3.2. Solid-State Characterization

#### 3.2.1. TGA

**Figure 6:**
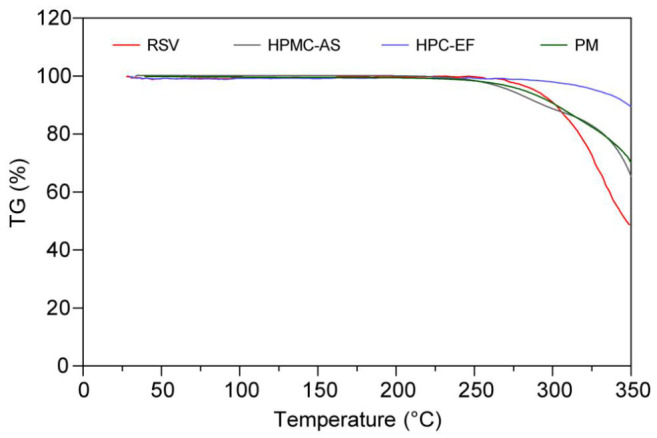
Thermogravimetric curves of RSV, HPMC-AS, HPC-EF, and the physical mixture (PM). The TG profiles show the percentage weight remaining as a function of temperature, with the main weight loss occurring above the selected processing temperature.

Thermal stability is critical for HME and FDM because both processes expose materials to elevated temperatures. The raw materials and PM were analyzed by TGA up to 350 °C. As shown in Figure 6, RSV began to degrade at approximately 250 °C, while HPMC-AS and HPC-EF showed major degradation events at approximately 250 °C and 300 °C, respectively. The PM showed a single-stage degradation profile beginning at approximately 250 °C, indicating acceptable thermal compatibility among RSV, HPMC-AS, and HPC-EF under the selected processing conditions. Because the FDM printing temperature was set to 220 °C, below the main degradation temperature of the components, the thermal risk during printing was considered limited.

#### 3.2.2. DSC

**Figure 7:**
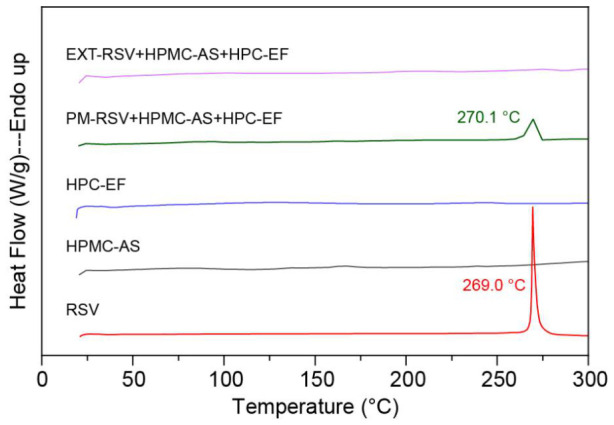
DSC thermograms of RSV, HPMC-AS, HPC-EF, the PM, and the EXT. The endothermic direction is indicated on the y-axis, and the labeled temperatures correspond to the observed thermal events in RSV and PM.

DSC was used to evaluate the thermal transitions and crystalline state of RSV in the raw materials, PM, and EXT. As shown in Figure 7, crystalline RSV exhibited a distinct endothermic melting peak at 269.0 °C, consistent with the reported melting point [50]. The PM showed a much weaker endothermic peak than pure RSV (47.3 mW/mg vs. 3.76 mW/mg), suggesting partial dissolution of RSV into the polymer matrix during heating before the remaining crystalline fraction melted near 269.0 °C. This behavior indicates good miscibility between RSV and the polymeric excipients during thermal processing.

The EXT showed no distinct RSV melting peak during heating, suggesting that RSV was dissolved or amorphously dispersed in the polymer matrix during HME. This result supports the formation of an ASD, although DSC alone cannot fully exclude the presence of a small residual crystalline fraction because of instrumental sensitivity and detection limits. Therefore, PXRD and PLM were further used to confirm the solid state of RSV in the extrudate.

#### 3.2.3. Hot-Staged PLM

**Figure 8:**
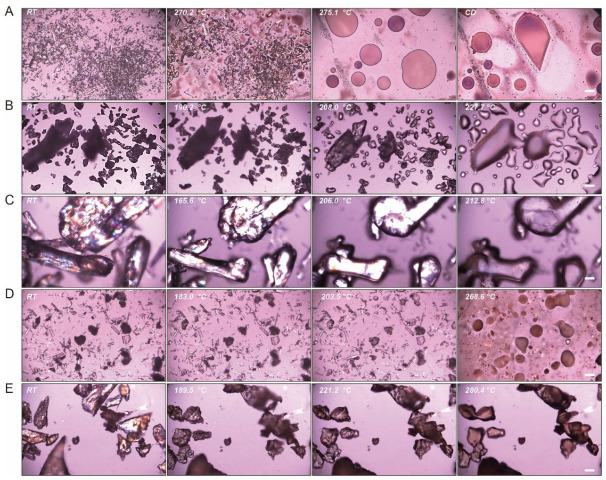
Hot-stage PLM images of each formulation during heating: (**A**) RSV; (**B**) HPMC-AS; (**C**) HPC-EF; (**D**) PM; (**E**) EXT. Scale bar = 100 μm.

Hot-stage PLM was used to monitor crystalline phase changes during heating. As shown in Figure 8A, RSV displayed clear birefringence under polarized light at room temperature, confirming its crystalline nature. RSV began to melt at approximately 270.2 °C and completed melting at approximately 275.1 °C. HPMC-AS and HPC-EF showed amorphous characteristics and softened at approximately 190.2 °C and 165.6 °C, respectively (Figure 8B,C). In the PM, polymer softening above approximately 203.5 °C allowed RSV to disperse into the polymer matrix, while residual crystalline RSV melted near 270 °C (Figure 8D). This indicates partial ASD formation under thermal treatment but incomplete conversion in the absence of strong mixing. By contrast, the EXT showed no birefringence, indicating that RSV was converted to an amorphous state within the polymer matrix (Figure 8E). Upon heating to approximately 280 °C, the EXT softened as a viscous matrix rather than showing the high-fluidity melting behavior of crystalline RSV. Together with the DSC results, the PLM observations support the formation of RSV-loaded ASDs during HME.

#### 3.2.4. PXRD

**Figure 9:**
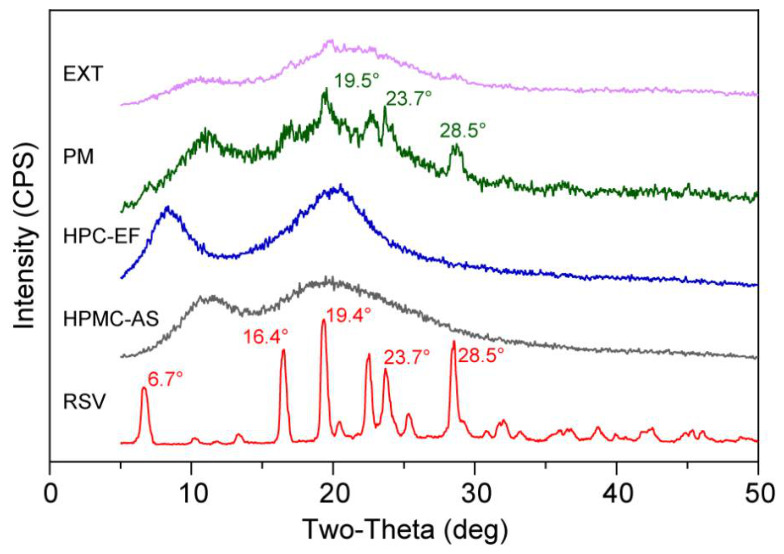
PXRD patterns of RSV, HPMC-AS, HPC-EF, the PM, and the EXT. The labeled diffraction angles indicate the selected characteristic peaks observed in the RSV and PM patterns.

PXRD was used to directly evaluate the crystalline state of RSV after HME. As shown in Figure 9, pure RSV exhibited characteristic diffraction peaks at 6.7°, 16.4°, 19.4°, 23.7°, and 28.5° (2θ). These peaks were also present in the PM, confirming that RSV remained crystalline after physical mixing. In contrast, the characteristic RSV peaks disappeared in the EXT, demonstrating that RSV was amorphously dispersed in the polymer matrix after HME. The combined DSC, PLM, and PXRD results confirm the successful formation of an RSV-loaded ASD.

### 3.3. FDM-Printed Tablets

The spatial control and layer-by-layer assembly enabled by FDM printing allow complex internal geometries to be fabricated with high precision. Previous studies have shown that tablet dimensions and shell layers can affect the release kinetics of active pharmaceutical ingredients [51]. In the present study, the comparison was narrowed to internal infill pattern while keeping the external dimensions, nominal dose, and formulation constant. Thus, the analysis focuses on whether pattern selection can modulate mechanical response and release within a fixed non-disintegrating polymer matrix, rather than claiming that infill pattern alone determines dissolution.

Eight internal patterns were initially designed, and seven were successfully fabricated by FDM printing. The concentric pattern (T4) could not be printed at the designed infill percentage because the circular internal lines were disconnected and structurally unstable; therefore, T4 was excluded from subsequent analysis. This result highlights the importance of manufacturability when selecting internal patterns for pharmaceutical printing.

**Figure 10:**
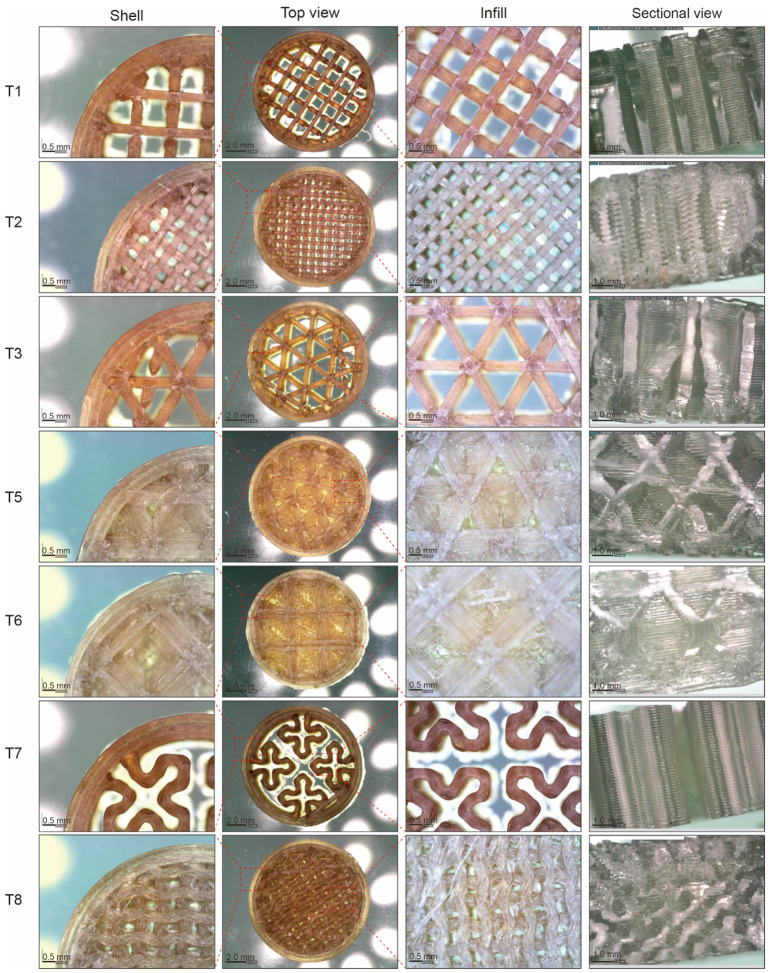
Structural diagrams of FDM-printed tablets with different internal infill patterns. Representative optical microscopic images of the shell, top view, infill region, and sectional view of tablets with different internal architectures (T1–T3 and T5–T8). Scale bars: shell, 0.5 mm; top view, 2.0 mm; infill, 0.5 mm; sectional view, 1.0 mm.

As shown in Figure 10, tablets T1–T3 and T5–T8 were fabricated with identical shell settings and external dimensions. The measured diameters ranged from 12.01 ± 0.06 to 12.09 ± 0.04 mm, with variation coefficients below 0.75%. Heights deviated from the design by 0.6–2.6%. The printed dimensions were slightly larger than the digital model, most likely because of radial expansion during cooling and solidification. Tablet weight and density were broadly consistent among patterns, indicating that the subsequent differences in mechanical properties and dissolution behavior were associated mainly with internal geometry, although small variations in strand arrangement, pore accessibility, and matrix hydration could also contribute.

To further support the qualitative structural observations, a quantitative image-based analysis of the internal infill architectures was performed (Table 3). The top-view accessible pore fraction and accessible pore area were calculated by excluding the 0.8 mm shell region from the measured tablet radius. As summarized in Table 3, the accessible pore fraction varied markedly among different infill patterns. T1 and T7 showed the highest accessible pore fractions, indicating more open and directly accessible pore networks. T3 showed an intermediate accessible pore fraction, whereas T2 showed a lower value, probably because of its smaller pore size despite the presence of numerous channels. In contrast, T5, T6, and T8 showed very low top-view accessible pore fractions, suggesting that their internal channels were less directly exposed to the external medium from the tablet surface. These quantitative descriptors support the visual observation that simple and open infill patterns provide more accessible entry points for medium penetration, whereas complex patterns restrict initial medium access.

### 3.4. Mechanical Properties of FDM-Printed Tablets

**Figure 11:**
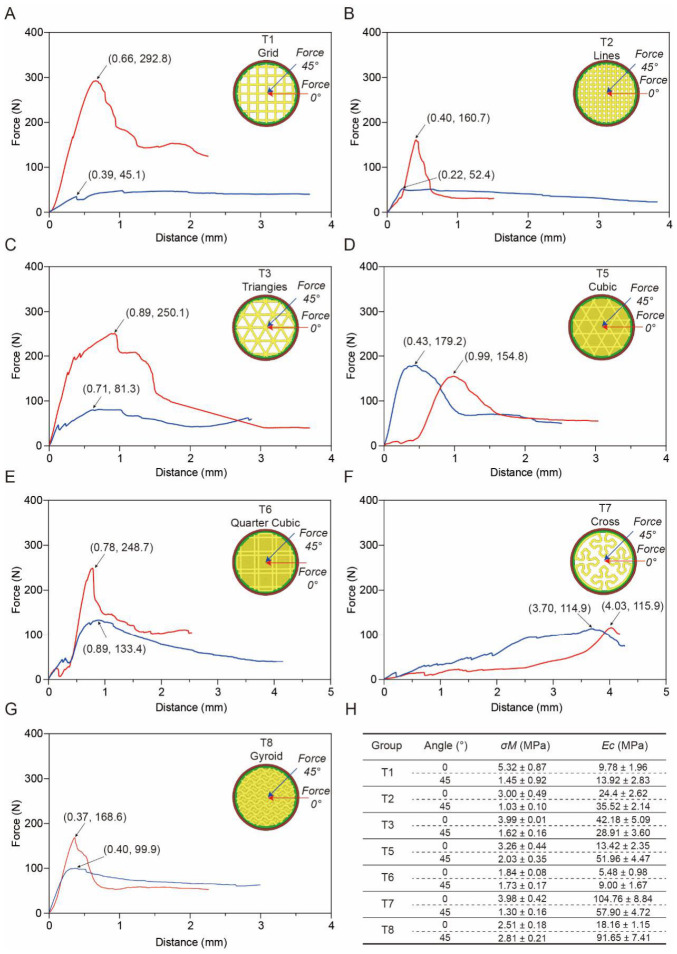
Force–distance curves and mechanical characterization of FDM-printed tablets under different loading orientations. Representative force–distance curves are shown for clarity. (**A**) Representative force–distance curves of T1 tablets with a grid infill pattern; (**B**) T2 tablets with a lines infill pattern; (**C**) T3 tablets with a triangles infill pattern; (**D**) T5 tablets with a cubic infill pattern; (**E**) T6 tablets with a quarter cubic infill pattern; (**F**) T7 tablets with a cross infill pattern; (**G**) T8 tablets with a gyroid infill pattern; (**H**) quantitative mechanical parameters, including maximum stress and compressive modulus. Quantitative mechanical parameters, including maximum stress and compressive modulus, are presented as mean ± SD (*n* = 3).

Compression testing was used to evaluate the mechanical response of the FDM-printed tablets. Compressive modulus was selected as a key parameter because it accounts for differences in specimen dimensions and reflects resistance to deformation. Peak force was used to indicate the maximum load-bearing capacity before failure. As shown in Figure 11, internal infill pattern influenced tablet mechanical performance. Most patterns showed mechanical anisotropy under 0° and 45° loading, and the degree of anisotropy was related to structural symmetry and load-bearing pathways. Directional patterns such as T1, T2, and T3 tended to show stronger load-bearing capacity when the loading direction aligned with the principal structural direction. The cross pattern (T7), with higher geometric symmetry, showed a more balanced response under both loading conditions. T5 and T8 exhibited distinct responses: T5 showed a higher modulus under 45° loading than under 0° loading, whereas T8 displayed the weakest overall mechanical performance. These results indicate that pattern complexity, periodicity, and symmetry can regulate stress transmission within printed polymer matrices.

Fracture distance, an indicator of deformation tolerance before failure, also depended on the infill pattern and loading orientation. For T1–T3, the main load-bearing pathways were more stable under 0° loading, resulting in shorter fracture distances. For example, T1 fractured at approximately 0.8 mm under 0° loading but at approximately 1.5 mm under 45° loading, indicating greater shear deformation when the loading direction deviated from the structural axis. Similar trends were observed for T2 and T3. In contrast, T7 distributed stress more uniformly and showed similar fracture distances of approximately 1.1 mm under both loading angles. T5 showed a reduced fracture distance under 45° loading because of its angle-dependent rigidity, whereas the continuous porous architecture of T8 allowed substantial plastic deformation, resulting in fracture distances exceeding 2.0 mm under both loading conditions.

The compressive modulus also varied among patterns and showed angle dependence. This variation reflects the combined influence of structural symmetry, internal load-bearing pathways, and alignment between the loading direction and the principal pattern direction. Symmetric structures maintained relatively stable modulus values across loading orientations, whereas directional structures showed larger changes when the loading angle shifted. For example, T1 showed modulus values of 9.8 MPa at 0° and 13.9 MPa at 45°, whereas T8 increased from 18.2 MPa at 0° to 91.7 MPa at 45°. T7 decreased from 104.8 MPa at 0° to 57.9 MPa at 45°. These findings demonstrate that internal pattern selection provides a practical means of tuning, but not independently predicting, the mechanical anisotropy and deformation behavior of FDM-printed tablets.

### 3.5. In Vitro Drug Release Studies

#### 3.5.1. Drug Release Profiles

**Figure 12:**
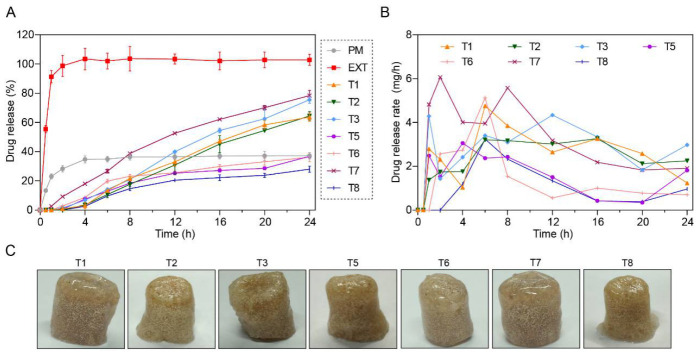
Drug release profiles of capsules (PM and EXT) and FDM 3D-printed tablets. (**A**) Cumulative drug release profiles of PM, EXT, and FDM 3D-printed tablets with different infill patterns; (**B**) drug release rates of FDM 3D-printed tablets with different infill patterns over 24 h; (**C**) representative optical images of FDM 3D-printed tablets with different infill patterns. Dissolution tests were performed in triplicate, and cumulative release data are presented as mean ± SD (*n* = 3).

The in vitro release profiles showed that FDM-printed tablets with different infill patterns released RSV differently from PM and EXT capsules. As shown in Figure 12, the PM and EXT capsules rapidly settled at the bottom of the dissolution vessel. Approximately 98% of RSV was released from the EXT capsules within 1 h, representing the fastest release among all groups. This rapid release is attributed to the absence of a fixed tablet structure after capsule dissolution, which increased the contact area between the ASD powder and the dissolution medium, together with the enhanced apparent solubility of RSV in the amorphous formulation. PM capsules also released RSV rapidly, reaching an apparent plateau within 4 h, but the final released amount was lower than that of EXT because RSV remained crystalline in the PM. These control results confirm that the printed tablet structure, rather than ASD formation alone, substantially slowed RSV release.

Because the densities of the printed tablets were lower than that of water (Table 4), all printed tablets initially floated on the dissolution medium. T7 sank after approximately 0.5 h, T1–T3 sank after approximately 2 h, whereas the more complex-patterned tablets remained floating for more than 4 h. The sinking behavior was associated with post-dissolution swelling: T7 showed the most extensive swelling, T1–T3 showed marked expansion, and tablets with more complex patterns showed limited swelling. Faster sinking may increase mechanical interaction with the medium and promote hydration of the internal polymer matrix.

The quantitative structural analysis further supported the observed release differences. T7 showed a high top-view accessible pore fraction and accessible pore area, which could facilitate rapid medium penetration, hydration, and swelling of the polymer matrix. T1 also exhibited a high accessible pore fraction, while T3 showed an intermediate value, consistent with their relatively continuous release behavior. Although T2 had a lower accessible pore fraction than T1 and T3, its dense line pattern still provided multiple narrow channels for medium entry. In contrast, T5, T6, and T8 exhibited very low top-view accessible pore fractions, indicating that the dissolution medium had fewer directly accessible entry points from the tablet surface. This restricted initial medium access may contribute to delayed hydration and slower RSV release.

Compared with EXT powder, the solid printed structures delayed RSV release because the dissolution medium had to penetrate the internal channels before drug diffusion could occur. T7, which had a simple and open porous architecture, exhibited the fastest release among the printed tablets. T1, T2, and T3 also possessed relatively simple through-channel structures and therefore showed release profiles close to that of T7. In contrast, T5, T6, and T8 contained more tortuous internal pathways, which restricted medium penetration and slowed drug release. These results indicate that the release behavior was influenced not only by the nominal infill percentage but also by the accessibility and arrangement of the internal pore network.

Table 4 shows that the printed tablets had comparable dimensions, weights, and densities; therefore, the differences in release behavior were mainly related to internal infill geometry. As shown in Figure 12, T1–T3 and T7 released more RSV at 24 h than T5, T6, and T8. However, none of the printed tablets achieved complete release within 24 h, and several patterns released only a limited fraction of the dose. Considering the finite residence time of oral dosage forms in the small intestine, this incomplete release indicates that the current HPMC-AS-based formulation should not be presented as an optimized RSV dosage form for complete intestinal absorption. Instead, the data support a more limited conclusion: infill pattern can modulate release from a fixed, non-disintegrating polymer matrix. Moreover, the top-view accessible pore fraction should be interpreted as a projected structural descriptor rather than a complete three-dimensional pore-network parameter. The relationship between pore architecture and drug release should also be interpreted cautiously. Once the pores and channels are hydrated, the external hydrodynamic boundary layer around the tablet and the effective diffusion path through the swollen matrix may reduce the influence of local pore size. Thus, the observed profiles reflect coupled effects of surface-accessible pores, medium penetration, swelling, diffusion-path tortuosity, and boundary-layer-controlled dissolution rather than a simple one-to-one correlation between infill pattern and release rate. Further quantitative measurements of three-dimensional pore connectivity, water uptake, erosion, and biorelevant gastrointestinal transit are needed to confirm the detailed mechanism.

**Table 4:** Geometric characteristics of FDM-printed tablets with different internal patterns.

#	Infill Pattern	Diameter (mm)	* Variation %	Height (mm)	* Variation %	Weight (mg)	** Variation %	Density (mg/mm^3^)
T1	Grid	12.05 ± 0.02	0.42	5.06 ± 0.15	1.2	420.46 ± 15.11	3.59	0.73
T2	Lines	12.09 ± 0.04	0.75	5.08 ± 0.19	1.6	416.47 ± 14.50	3.48	0.71
T3	Triangles	12.04 ± 0.03	0.33	5.11 ± 0.15	2.2	415.48 ± 8.37	2.01	0.71
T5	Cubic	12.04 ± 0.02	0.33	5.00 ± 0.09	0.6	410.58 ± 19.91	4.86	0.71
T6	Quarter Cubic	12.07 ± 0.03	0.58	5.07 ± 0.13	1.4	427.05 ± 12.97	3.04	0.74
T7	Cross	12.01 ± 0.06	0.08	5.13 ± 0.12	2.6	411.54 ± 4.72	1.15	0.71
T8	Gyroid	12.05 ± 0.02	0.42	5.08 ± 0.09	1.6	426.19 ± 13.14	3.08	0.74

* *The variation in diameter and height were calculated using following equation:*

$$\;Variation\;\%=\frac{D_{m}-D_{d}}{D_{d}}*100\%$$

*where* $D_{m}$ *is the measured diameter or height of the printed tablets, while* $D_{d}$ *is the designed diameter or height of the digital tablet models.*

** *the variation in weight was calculated using following equation:*

$$Variation\;\%=\frac{S.D.}{Average}*100\%$$

*where* S.D. *is the standard error of all six measure tablets, while* Average *is the average value of all six measure tablets.*

#### 3.5.2. Release-Kinetic Analysis

To clarify and compare the release behavior of each dosage form, the dissolution data were fitted using the models listed in method. For PM, the low R^2^ values for the zero-order (0.6666) and first-order (0.7183) models indicate that neither simple model adequately described its release. The higher Korsmeyer–Peppas R^2^ value (0.9601) suggests that PM release involved a combination of dissolution and diffusion processes. For EXT, the first-order model provided the best fit (R^2^ = 0.9966), indicating concentration-dependent release from the dispersed ASD powder after capsule dissolution. These model assignments were used for comparative interpretation and should not be considered definitive proof of a single release mechanism (Table 5).

T7 also showed rapid release and was best fitted by the first-order model (R^2^ = 0.9987), with good fitting by the Korsmeyer–Peppas model (R^2^ = 0.9946). This behavior is consistent with its open internal structure, high top-view accessible pore fraction, extensive swelling, and rapid medium penetration. Therefore, unlike the more gradual release observed for T1–T3, T7 behaved as a faster-releasing printed structure whose release rate depended strongly on remaining drug content and rapid hydration of the matrix.

T1, T2, and T3 showed strong zero-order fitting, with R^2^ values of 0.9932, 0.9946, and 0.9959, respectively. These results indicate more uniform release over the tested period, although the release remained incomplete at 24 h. The Korsmeyer–Peppas model also provided good fits, with n values of 1.124, 1.225, and 1.141 for T1, T2, and T3, respectively. Values above 1 are commonly associated with super Case-II transport, suggesting that polymer-chain relaxation and swelling contributed to RSV release [25]. The Peppas–Sahlin model showed m values of 0.89 for these tablets, further supporting the role of polymer relaxation relative to purely Fickian diffusion [52]. The relatively simple but structurally stable internal channels of T1–T3 likely enabled continuous hydration and swelling, resulting in near zero-order release behavior within this in vitro setting. The structural descriptors further indicated that T1 and T3 had relatively high accessible pore areas, which may support continuous medium access during dissolution. Although T2 showed a lower top-view accessible pore fraction, its dense line pattern may still provide multiple narrow channels for gradual medium penetration, contributing to its controlled-release behavior.

T5, T6, and T8 exhibited slower release, lower release constants, and acceptable but less mechanistically distinctive fits across several models. Their Korsmeyer–Peppas n values ranged from 0.682 to 0.853, and their Peppas–Sahlin k2 values were below 0.001, suggesting that diffusion through restricted channels dominated while the contribution of polymer relaxation or erosion was limited. These kinetic results were consistent with both the dissolution profiles and the quantitative structural analysis. The complex internal patterns showed very low top-view accessible pore fractions, suggesting fewer directly exposed medium-entry points and delayed development of effective diffusion pathways. This restricted medium access was also consistent with the limited swelling observed for these tablets, thereby contributing to slower RSV release. Nevertheless, because these tablets released only a relatively low fraction of RSV within 24 h, the kinetic parameters should be interpreted mainly as indicators of relative release trends rather than as evidence of clinically adequate delivery.

Overall, internal structure should be selected according to the intended release profile and polymer matrix. Highly open structures such as T7 may be suitable when faster release from this matrix is desired. Moderately regular structures such as T1–T3 are more appropriate for controlled-release designs requiring more predictable release over the tested interval. More complex structures such as T5, T6, and T8 can slow drug release when prolonged diffusion-controlled behavior is desired; however, for RSV, the incomplete release observed here suggests that future work should evaluate faster-dissolving or blended polymer systems, optimize HPMC-AS grade and content, and include biorelevant dissolution conditions before individualized product performance is claimed. In addition, future studies should include three-dimensional structural characterization, such as micro-CT analysis, to more accurately quantify pore connectivity, specific surface area, and tortuosity within the whole tablet.

## 4. Conclusions

This study developed RSV-loaded FDM-printed tablets by combining HME-based ASD preparation with digital infill-pattern design. TGA confirmed that the selected formulation was thermally stable under the 220 °C printing condition. HPMC-AS and HPC-EF formed a printable polymer matrix that enhanced RSV apparent solubility, and DSC, PLM, and PXRD confirmed that RSV was converted from a crystalline state to an amorphous state during HME. The printed tablets showed high dimensional reproducibility, with diameter and height variations below 2.6% and weight variation coefficients below 4.86%.

The main finding of this work is that internal infill pattern can modulate, within the limits of a fixed HPMC-AS/HPC-EF matrix, the mechanical and in vitro release performance of FDM-printed polymeric tablets. In addition to qualitative microscopic observation, image-based quantitative structural descriptors were introduced to compare the different infill architectures. The top-view accessible pore fraction and accessible pore area varied markedly among patterns. T1 and T7 showed the highest accessible pore fractions, whereas T5, T6, and T8 showed much lower values. These results provide quantitative support for the interpretation that open and directly accessible channels promote medium penetration and hydration, whereas complex internal architectures restrict initial medium access.

Directional and regular patterns such as T1–T3 showed stronger load-bearing behavior and clear mechanical anisotropy. The symmetric cross pattern (T7) produced a more balanced mechanical response and faster release because of its open structure and extensive swelling. The high accessible pore fraction of T7 further supports its rapid medium penetration and fast release behavior. More complex patterns such as T5, T6, and T8 restricted medium penetration, reduced swelling, and delayed RSV release through more tortuous diffusion pathways. Their low top-view accessible pore fractions suggest fewer directly exposed medium-entry points. Kinetic analysis further showed that T1–T3 achieved near zero-order release within the test period, T7 followed rapid first-order release, and complex patterns were associated with slower diffusion-restricted release.

These results support the use of polymer-matrix and infill-pattern coupling as a strategy for tuning FDM-printed oral dosage forms without changing nominal dose or composition. However, the present tablets did not achieve complete RSV release within 24 h, and the enteric pH-dependent nature of HPMC-AS may limit release during the available intestinal residence time. Therefore, this study should be positioned as a mechanistic investigation of geometry-dependent modulation rather than a completed RSV product-development study. Future work should specify and optimize the HPMC-AS grade, evaluate faster-dissolving or mixed-polymer matrices, quantify pore connectivity and water uptake, and test release under biorelevant gastrointestinal conditions. In particular, three-dimensional imaging methods such as micro-CT should be used in future studies to obtain more comprehensive structural parameters, including true porosity, specific surface area, pore connectivity, and tortuosity.

## Figures and Tables

**Table 1:** HME formulations containing RSV, HPMC-AS, and HPC-EF at different mass ratios.

Formulation	Ratio of RSV	Ratio of HPMC-AS	Ratio of HPC-EF
1	20	80	0
2	20	78	2
3	20	75	5
4	20	70	10

**Table 2:** The formula and definition of different mathematical models.

Mathematical Models	Formula	Definition
Zero-order	$Q_{t}=Q_{0}+K_{0}\times t$	Q_t_: the amount of drug released in time t Q_0_: the initial amount of drug in the solution K_0_: the zero-order release constant
First-order	$\log C=\log C_{0}-kt/2.303$	C: the amount of drug at time t $C_{0}$: the initial concentration k: the first order rate constant
Higuchi	$\frac{Q_{t}}{Q_{\infty }}=k{\times t}^{1/2}$	Q_t_: the drug released at time t $Q_{\infty }$: the drug loading of the dosage K: Higuchi constant
Korsmeyer–Peppas	$\frac{Q_{t}}{Q_{\infty }}=k{\times t}^{n}$	Q_t_: the drug released at time t $Q_{\infty }$: the drug loading of the dosage k: the rate constant n: the release exponent
Peppas–Sahlin	$\frac{Q_{t}}{Q_{\infty }}=k_{1}{\times t}^{m}+k_{2}{\times t}^{2m}$	Q_t_: drug released at time t $Q_{\infty }:$ is the drug loading of the dosage k_1_ /k_2_: the kinetic constant m: the release exponent

**Table 3:** Quantitative structural descriptors of FDM-printed tablets with different infill patterns.

#	Infill Pattern	Internal Infill Area	Top-View Accessible Pore Fraction (%)	Accessible Pore Area (mm^2^)
		(mm^2^)		
T1	Grid	85.77	38.3	32.85
T2	Lines	86.40	13.4	11.58
T3	Triangles	85.59	23.8	20.37
T5	Cubic	85.59	0.0	0.00
T6	Quarter Cubic	86.08	0.6	0.52
T7	Cross	85.10	37.8	32.17
T8	Gyroid	85.77	0.1	0.09

Note: The internal infill area was calculated by excluding the designed shell thickness of 0.8 mm from the measured tablet radius. The top-view accessible pore fraction was obtained from optical microscopic images and represents a two-dimensional projected descriptor of visible surface-accessible pores, rather than true three-dimensional porosity.

**Table 5:** The kinetic constant and correlation coefficients of the different models.

#	Zero-Order		First-Order		Higuchi		Korsmeyer–Peppas			Peppas–Sahlin			
	K_0_	R^2^	k	R^2^	k	R^2^	k_kp_	n	R^2^	k_1_	k_2_	m	R^2^
PM	2.263	0.6666	0.033	0.7183	10.183	0.8322	23.366	0.173	0.9601	12.501	0	0.45	0.4338
EXT	6.391	0.5015	1.878	0.9966	29.413	0.6869	83.185	0.086	0.7376	37.049	0	0.45	0.2213
T1	2.744	0.9932	0.037	0.9880	10.433	0.9497	1.916	1.124	0.9924	2.874	0.037	0.89	0.9932
T2	2.636	0.9946	0.035	0.9849	9.939	0.9413	1.374	1.225	0.9954	2.311	0.056	0.89	0.9946
T3	3.316	0.9959	0.044	0.9885	11.923	0.9725	2.086	1.141	0.9947	3.166	0.048	0.89	0.9823
T5	1.635	0.9730	0.020	0.9822	6.455	0.9739	2.856	0.806	0.9829	2.859	<0.001	0.81	0.9629
T6	1.775	0.9490	0.022	0.9657	7.143	0.9751	4.417	0.682	0.9753	4.092	<0.001	0.71	0.9464
T7	3.653	0.9847	0.060	0.9987	14.484	0.9850	6.871	0.780	0.9946	6.874	<0.001	0.78	0.9878
T8	1.290	0.9686	0.015	0.9763	5.055	0.9637	1.969	0.853	0.9759	1.970	<0.001	0.85	0.9481

## Data Availability

The original contributions presented in this study are included in the article/Supplementary Materials. Further inquiries can be directed to the corresponding authors.

## Supplementary Materials

The following supporting information can be downloaded at: https://www.mdpi.com/article/10.3390/polym18121531/s1, Table S1. The formulation used for preliminary formulation screening studies. Table S2. Solubility of RSV formulations prepared by physical mixing (PM) and melt processing (MM) in pH 6.8 medium at 37 °C for 24 h. Figure S1. Results of calibration curves, correlation coefficients and linear ranges of RSV. Figure S2. Recovery of RSV after FDM 3D printing. Data are presented as mean ± SD (n = 3).
