# Supplementary material for: Infill Pattern-Dependent Mechanical Properties and In Vitro Release Behavior of FDM 3D-Printed Resveratrol Amorphous Solid Dispersion Matrix Tablets
Source: Polymers (Basel). 2026 Jun 19;18(12):1531. doi: 10.3390/polym18121531 (PMC13307023; doi:10.3390/polym18121531)
Supplement: Supplementary file 1 [file polymers-18-01531-s001.zip › polymers-4340047-supplementary.pdf]

Supplement information

Table S1. The formulation used for preliminary formulation screening studies.

Polymers	Structure
HPMC-AS	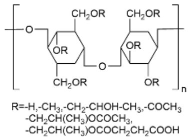
HPC-EF	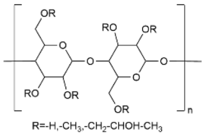
Soluplus®	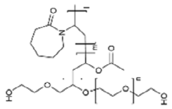
Plasdone K25 & Plasdone K90	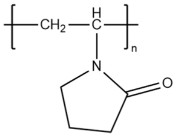
Kollidon® VA64	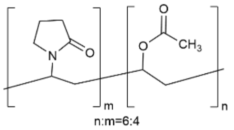
Kollidon® SR	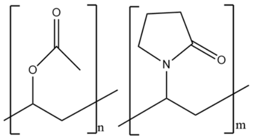

Table S2. Solubility of RSV formulations prepared by physical mixing (PM) and melt processing (MM) in pH 6.8 medium at 37 °C for 24 h.

Group	Solubility (μg/mL)
RSV	50.6
PM-K25+RSV	53.5
EXT-K25+RSV	105.0
PM-VA64+RSV	71.0
EXT-VA64+RSV	183.6
PM-K90+RSV	56.8
EXT-K90+RSV	83.9
PM-SR+RSV	54.4
EXT-SR+RSV	70.6
PM-Soluplus+RSV	104.3
EXT-Soluplus+RSV	236.2
PM-HPMC-AS+RSV	57.3
EXT-HPMC-AS+RSV	258.4
PM-HPC-EF+RSV	59.6
EXT-HPC-EF+RSV	127.6

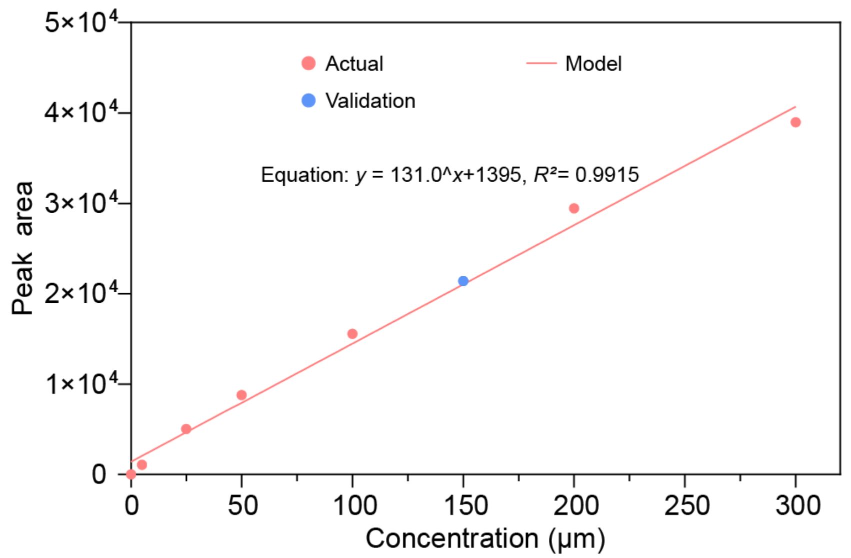

Figure S1. Results of calibration curves, correlation coefficients and linear ranges of RSV.

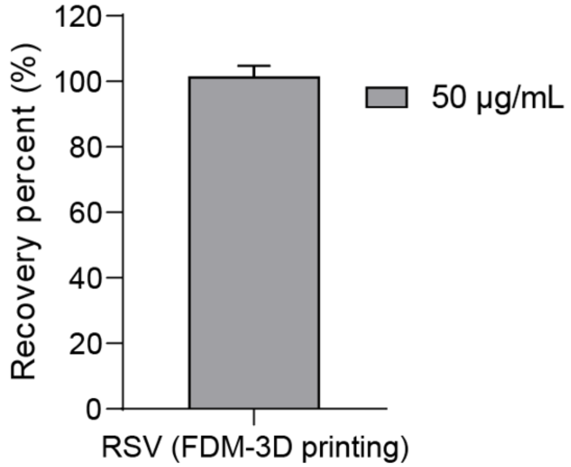

Figure S2. Recovery of RSV after FDM 3D printing.
Data are presented as mean ± SD (n = 3).
